# Supplementary material for: Immune Niche Formation in Engineered Mouse Models Reveals Mechanisms of Tumor Dormancy
Source: bioRxiv. 2025 Apr 18:2025.04.16.649000. Preprint. [Version 1] doi: 10.1101/2025.04.16.649000 (PMC12190759; doi:10.1101/2025.04.16.649000)
Supplement: Supplement 1 [file media-1.pdf]

Supplemental Fig. 1

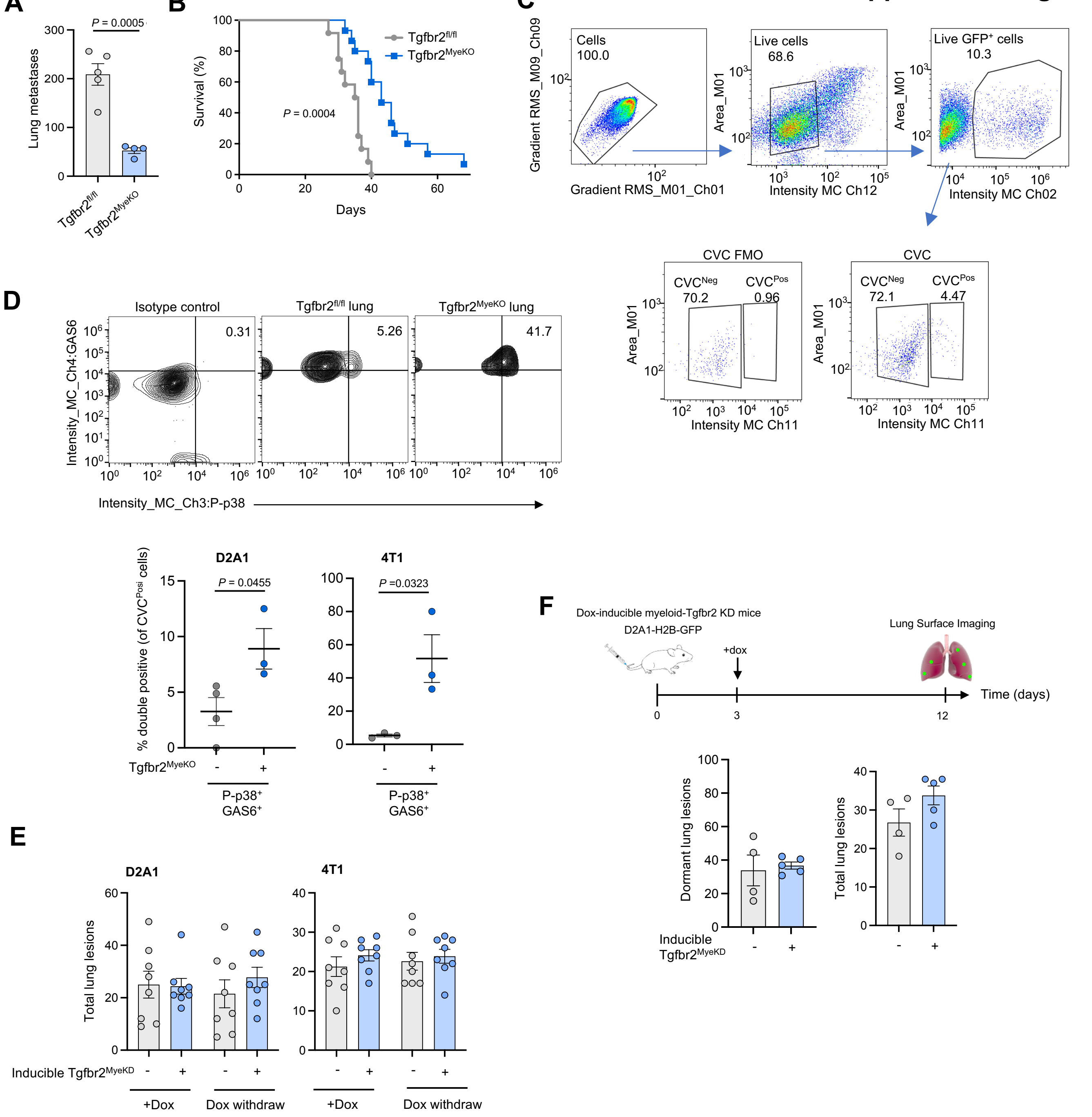

Figure S1. Tumor dormancy induction mediated by abrogation of myeloid-specific TGF-β signaling. A. Decreased lung metastasis in Tgfbr2^{MyeKO} mice compared to flox cont. mice that received tail vein injection (TVI) of D2A1 cells (n=4-5 mice per group). **B.** Tgfbr2^{MyeKO} mice survived longer than flox cont. after receiving TVI of D2A1 cells. Statistical analysis by Wilcoxon test. **C.** Imaging flow cytometry gating strategy for GFP^{+}/CVC^{Pos} D2A1 cells. **D.** Imaging flow gating strategy for P-p38 and GAS6 (left) and graphs showing more CVC^{Pos} D2A1 and 4T1 cells were P-p38 and GAS6 double positive from Tgfbr2^{MyeKO} mice than flox cont (n=3-4 mice per group). **E.** Total lung lesions did not change with myeloid Tgfbr2 KD or re-expression in the D2A1 TVI model (left) at and 4T1 orthotopic model (right) (n=8 mice per group). **F.** Dox-induced myeloid-Tgfbr2 KD after D2A1 TVI did not display a tumor dormancy phenotype suggesting the importance of a preestablished lung microenvironment (n=4-5 mice per group). Statistical analysis by unpaired two-tailed t-test. All error bars represent mean ± s.e.m.

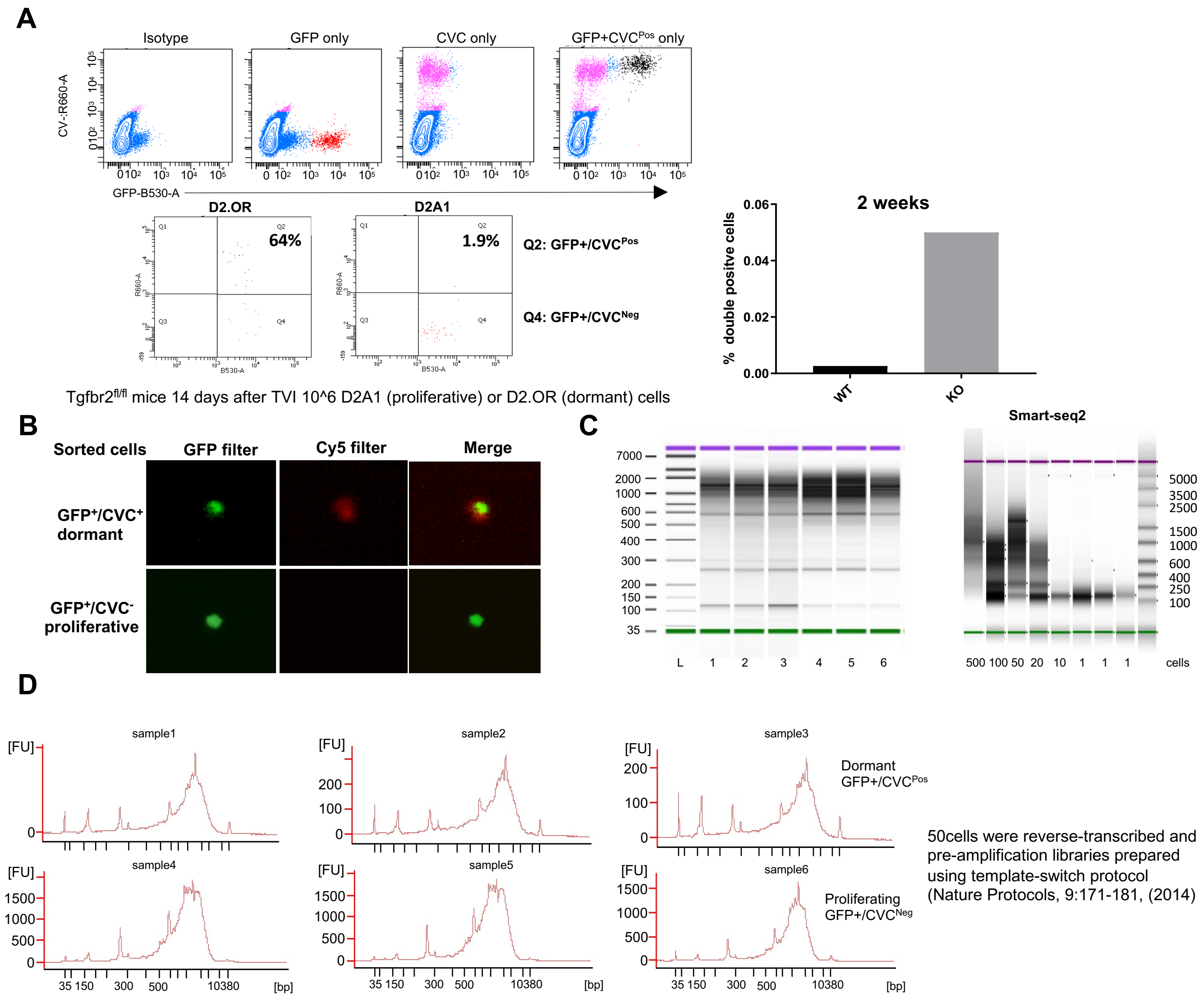

Figure S2. Identification of dormant tumor cells and RNA sequencing validation A. Flow cytometry plots for sorting dormant (GFP+CVC^{Pos}) vs proliferative (GFP+CVC^{Neg}) cancer cells before TVI (top panel) and flow cytometry gating of GFP+CVC^{Pos} D2.OR and D2A1 lung metastasis (bottom left panel) and quantification showing more GFP+CVC^{Pos} double positive D2A1 lung metastasis from Tgfbr2^{MyeKO} mice compared to flox cont. **B.** Sorting validation of GFP+CVC^{Pos} and GFP+CVC^{Neg} D2A1 cells **C.** RNA quality for sequencing. **D.** Pre-amplification libraries from dormant cells (n=50) after reverse transcription.

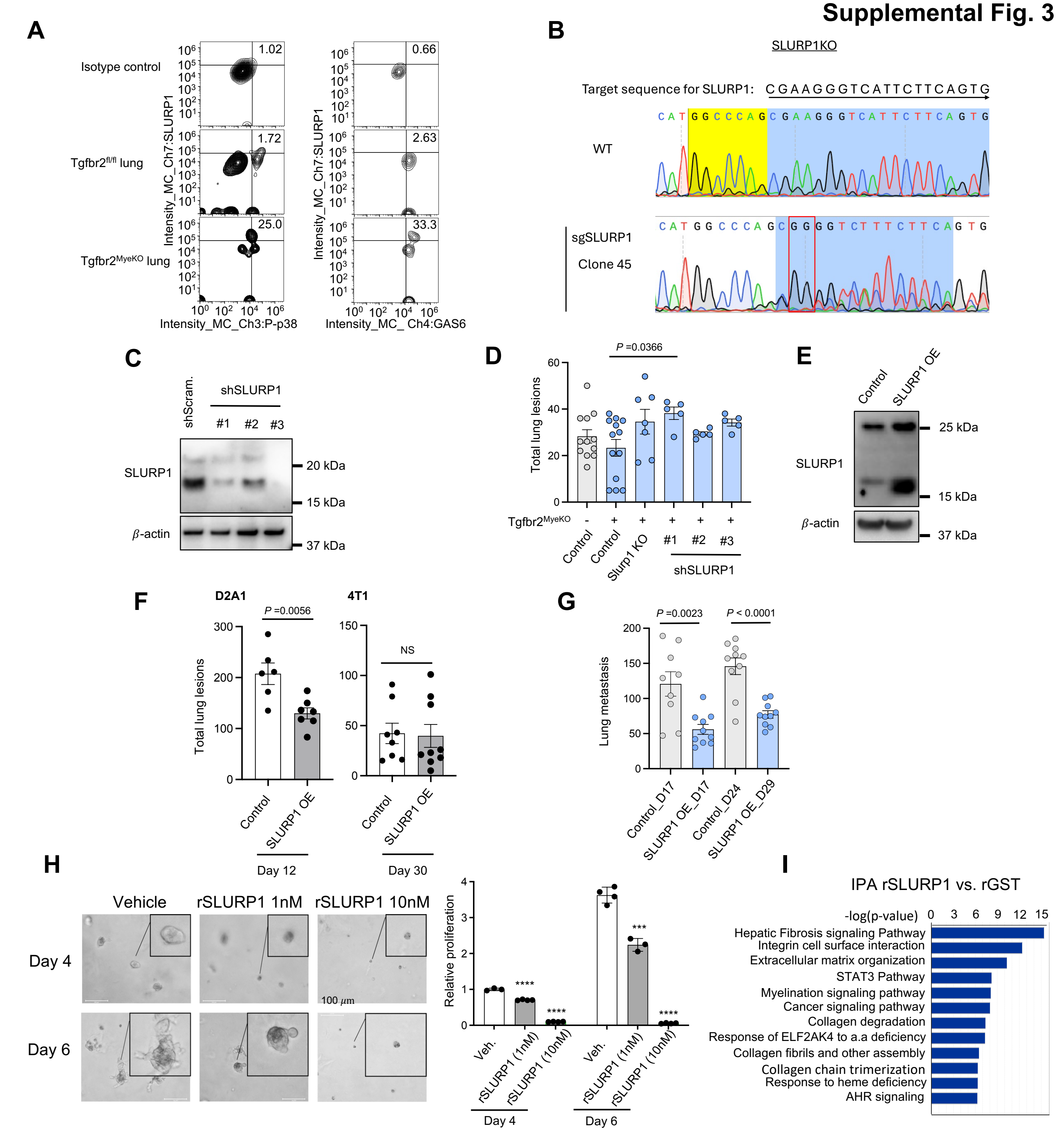

Figure S3. SLURP1 regulation of tumor cell proliferation and dormancy. A. Imaging flow cytometry gating for the expression of SLURP1, P-p38, and GAS6 in dormant (GFP+CVC^{Pos}) and proliferative (GFP+ CVC^{Neg}) D2A1 cells. **B.** Sanger sequencing chromatograms for Slurp1 KO clone. **C.** Western blot confirming KD of SLURP1 in D2A1 cells. **D.** Total tumor lesions mostly did not differ between Slurp1 KO and KD and control D2A1 cells in Tgfbr2^{MyeKO} mice (n=5-13 mice per group). **E.** Western blot of SLURP1 overexpression in 4T1 cells. **F.** Total lung lesions from D2A1 (left) and 4T1 cells (right) with SLURP1 overexpression **G.** Metastasis nodule count by Indian Ink staining from mice that received TVI of SLURP1 overexpressing D2A1 cells (n=9-10 mice per group). **H.** Recombinant SLURP1 inhibited D2A1 spheroid growth in a dose-dependent manner (left) and quantitative data (right) (n=3-4 biological replicates).**p<0.01, ***p<0.001, ****p<0.0001 **I.** IPA of rGST vs. rSLURP1 treated D2A1 cells in 3D culture. Statistical analysis by unpaired two-tailed t-test. All error bars represent mean ± s.e.m.

Supplemental Fig. 4

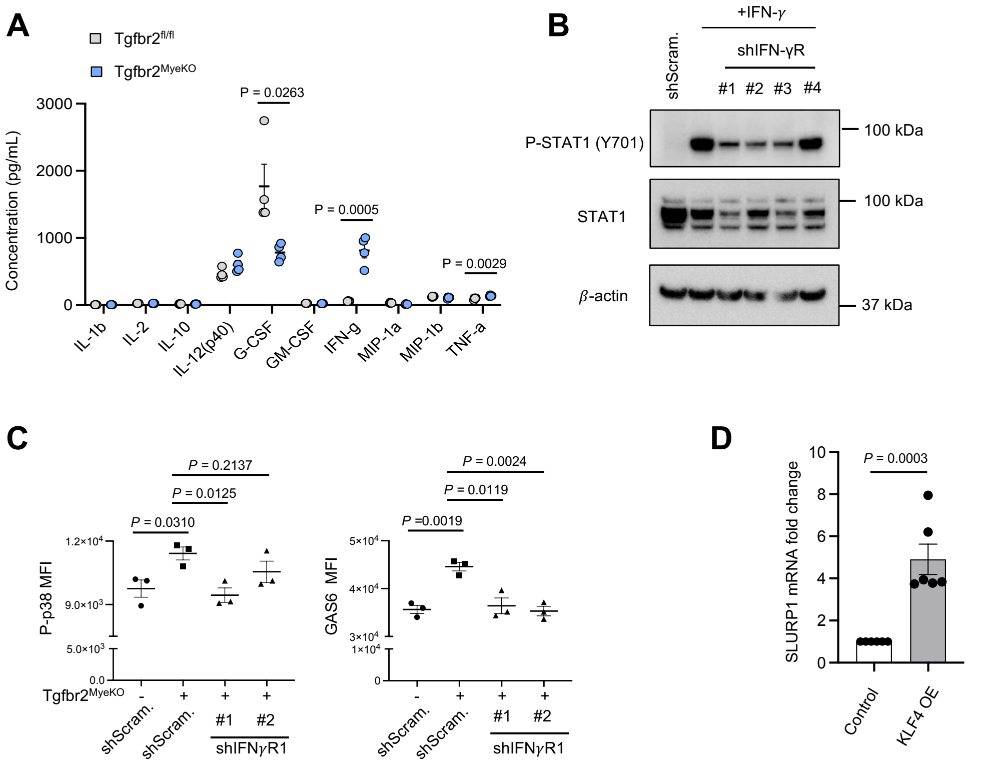

Figure S4. Mechanisms of SLURP1 regulation A. Bio-Plex Immunoassay showing increased IFN-γ and TNF-α in the lung lysate from tumor-bearing Tgfbr2^{MyeKO} mice (n=4 mice per group). **B.** Western blot confirming reduced STAT1 phosphorylation in shRNA IFN-γR KD cells following treatment with recombinant IFN-γ (100ng/mL) for 30 minutes. **C.** P-p38 and GAS6 expression from Imaging flow cytometry of dormant cells with or without IFN-γR KD, single cell suspension from the lungs of the tumor-bearing mice. n=3 biologically independent experiments. **D.** RT-qPCR showing over-expression of KLF4 increases SLURP1 mRNA in D2A1 cells (n=6 biological replicates). Statistical analysis by unpaired two-tailed t-test. All error bars represent mean $\pm$ s.e.m.

Supplemental Fig. 5

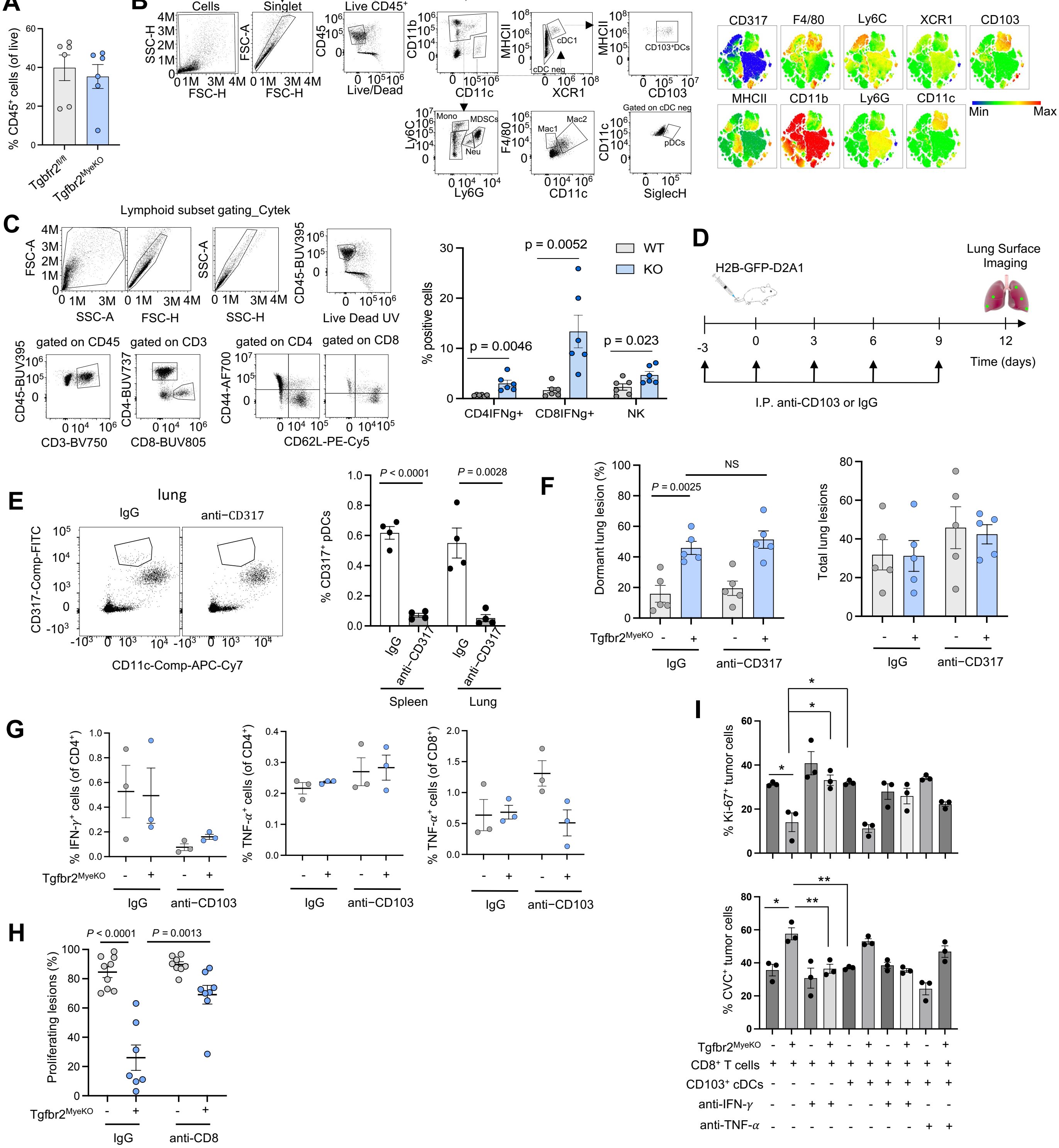

Figure S5. IFN-γ mediated immune surveillance in tumor dormancy. A. Cytek of the CD45⁺ cells from lungs of Tgfbr2MyeKO and flox cont. mice. **B**. Cytek gating strategy and tSNE plots for myeloid cell subclusters; the expression intensity for each marker is indicated by the color scale bar. **C.** Cytek gating strategy for lymphoid cell subsets (left). And % IFN-γ+ T cells and NK cells (right). **D.** Experimental design for the depletion of CD103⁺ cDCs. **E.** Gating strategy (left) and validation of CD317⁺ pDCs depletion in spleens and lungs of mice (right) (n=4 mice per group). **F.** No difference in dormant lung lesions upon CD317⁺ pDC depletion (n=5 mice per group). **G.** Flow cytometry analysis of IFN-γ and TNF-α expression in CD4+ T cells and TNF-α expression in CD8⁺ T cells upon CD103⁺ cDC depletion (n=3 mice per group). **H.** CD8⁺ T cell depletion increased proliferative lesions. **I.** Bar plot showing the percent of Ki-67⁺ (top) and CVCPos (bottom) D2A1 cells in the presence of CD8⁺ T cells or CD103⁺ cDCs or IFN-γ or TNF-α neutralizing antibodies (n=3 biological replicates). *p<0.5, **p<0.01. Statistical analysis by unpaired two-tailed t-test. All error bars represent mean ± s.e.m.

Supplemental Fig. 6

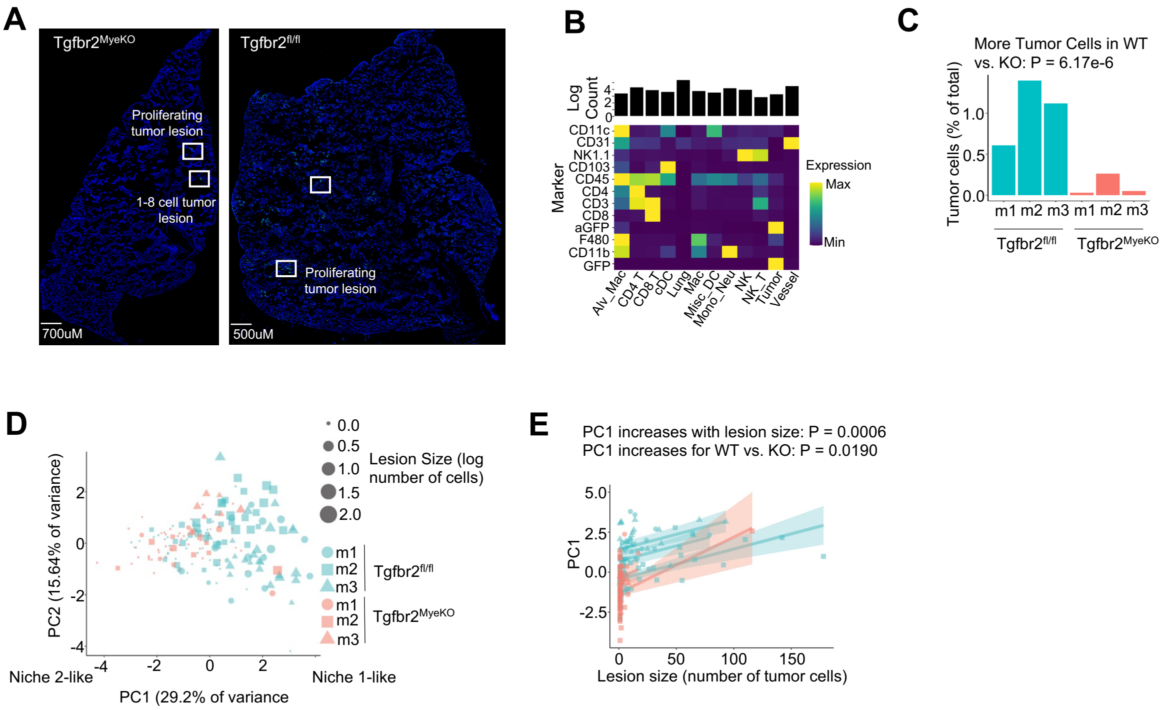

Figure S6. Characterization of immune niches for dormant and proliferative tumor lesions A. Whole lung tissue with representative imaging of tumor lesions. **B**. Clustering showing average cell markers profiles for the 12 known cell types identified from mouse lung IBEX images. **C**. Percentage of tumor cells in each mouse lung sample. Flox cont. mice contain more tumor cells (binomial regression model, P = 6.17 × 10-6). **D.** PCA of tumor lesions based on standardized CLRs for each immune cell type**. E.** PC1 scores for each tumor lesion as a function of lesion size and genotype. Smaller lesions and lesions from Tgfbr2^{MyeKO} mice have lower PC1 scores on average (Gaussian regression model, P = 0.0006 and P = 0.0190, respectively).

Supplemental Fig. 7

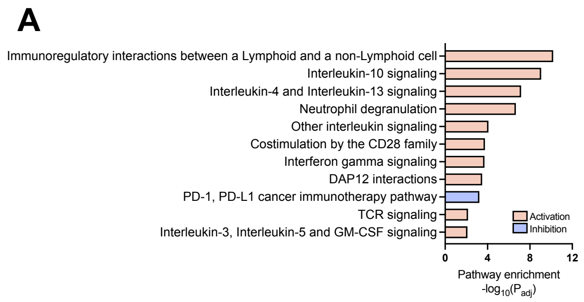

B

Pathway	Genes
Immunoregulatory interactions between a Lymphoid and a non-Lymphoid cell	c3,cd200,cd200r1,cd300c,cd300ld,cd300lf,cd3e,cd3g,cd40,cd8a, colec12,crtam,fcgr2b,fcgr3a/fcgr3b,hcst,itgb2,klrg1,lilra5,lilrb3, lilrb4, ncr1,pilrb,sftpd,sh2d1b,slamf6,trem1,trem2,tyrobp
Interleukin-10 signaling	ccl2,ccl4,ccl5,ccr1,ccr2,ccr5,cd86,fcer2,il10ra,il1rn,ptafr,ptgs2,tnf
Interleukin-4 and Interleukin-13 signaling	batf, ccl2, f13a1, fcer2, hgf, ighg, il13ra2, itgam, itgax, itgb2, ndn, osm, pik3r1, ptgs2, rora, s1pr1, tnf

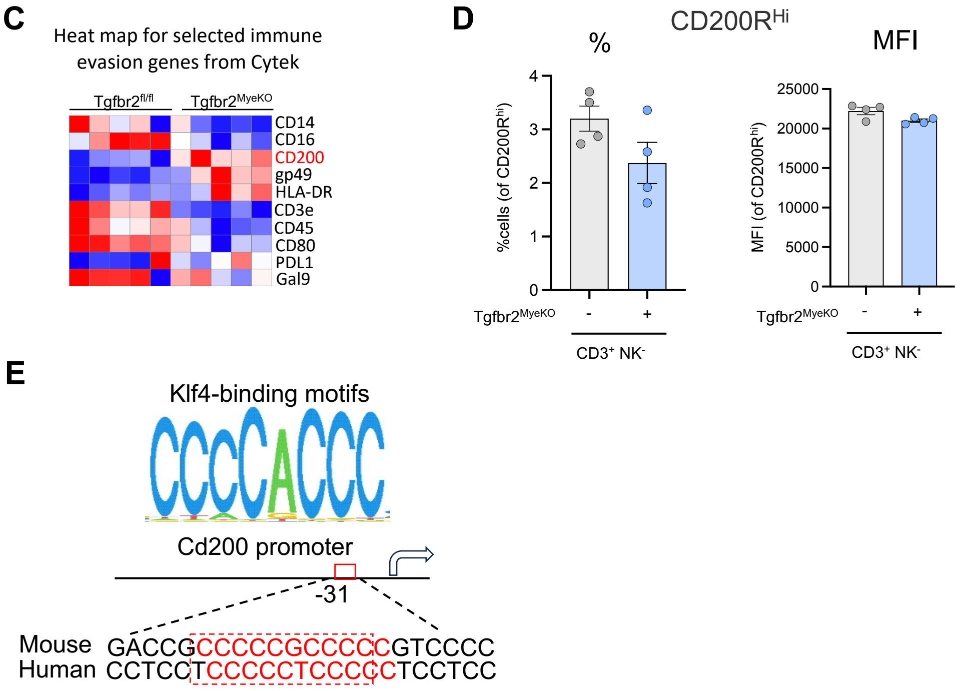

Figure S7. Immune evasion of dormant tumor cells. A. Pathway analysis from RNA-seq data comparing dormant vs. proliferative tumor cells indicating potential immune evasion mechanisms in tumor dormancy. **B.** Top gene candidates from the top three differential pathways and identification of CD200-CD200R1 regulatory axis. **C.** Heat map from Cytek analysis for CD200 validation among other immune modulatory genes. **D.** no difference in % $CD3^{+}CD200R^{Hi}$ T cells nor in $CD200R^{Hi}$ MFI comparing $Tgfbr2^{MyeKO}$ and flox cont. mice (n=4 mice per group). **E.** KLF4 binding site mapping in the *Cd200* promoter by Homer assay. KLF4 binding motif CCCCACCC was shown in mouse and human *CD200* promoters. Statistical analysis by unpaired two-tailed t-test. All error bars represent mean ± s.e.m.

Supplemental Fig. 8

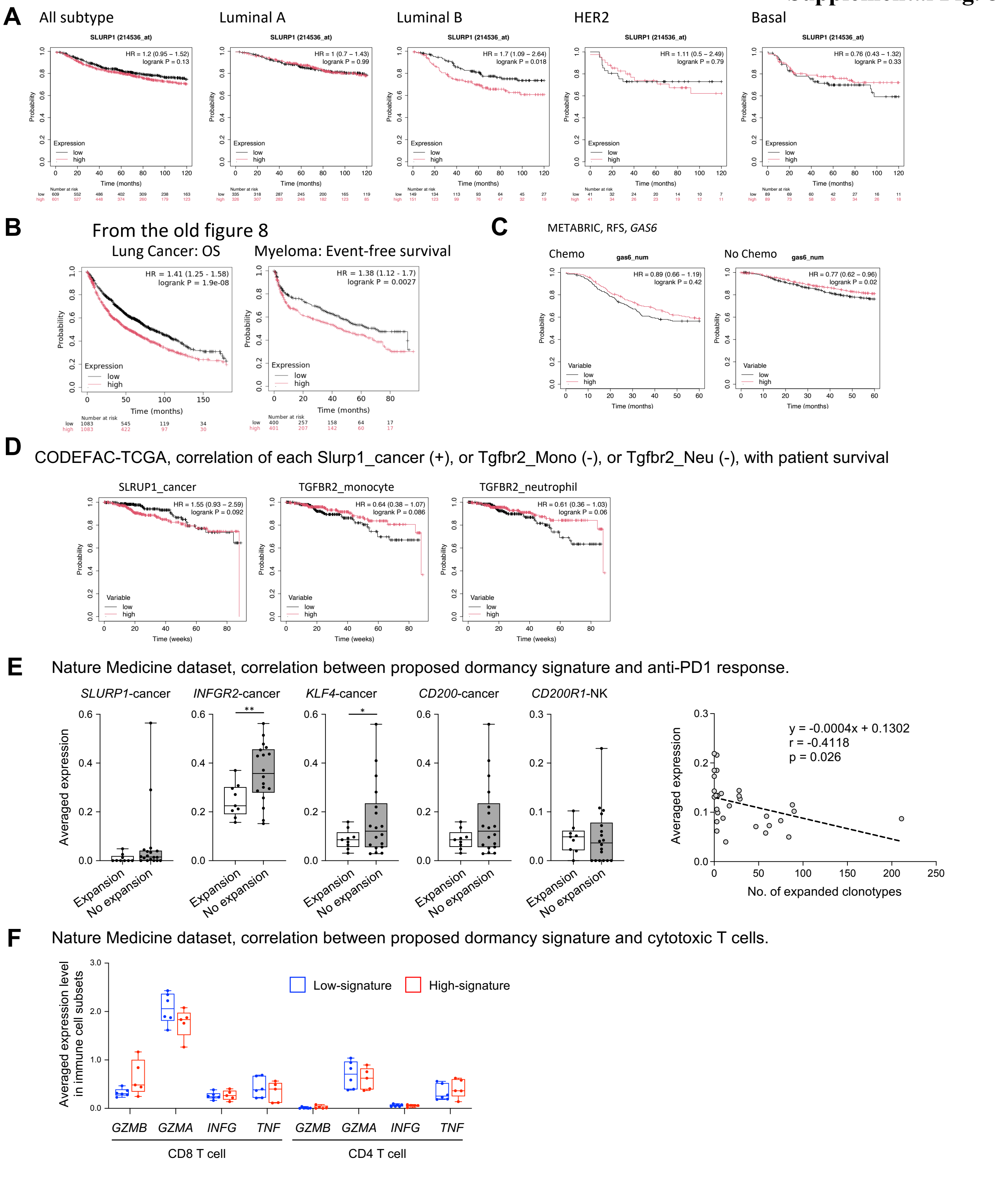

Figure S8. Human correlative studies of SLURP1, TβRII, and CD200-CD200R. A. High SLURP1 predicted a decreased DMFS for Luminal B patients (Earlier 2017, Exclude biased dataset) Q1 vs Q3, by KM-plotter. **B.** High SLURP1 predicted a decreased OS or Event free survival for lung cancer and myeloma patients, TCGA dataset. **C.** SLURP1 but not GAS6 predicted an increased RFS in breast cancer patients treated with chemotherapy compared with those untreated. **D.** The correlation of SLURP1 in cancer cells alone, or TβRII in monocytes alone, or TβRII in neutrophiles alone with patient survival in breast cancer patient dataset from TCGA CODEFACS, **E.** Average expression levels of *SLURP1*, *INGFR2*, *KLF4*, *CD200* from cancer cells and *CD200R1* from NK cells comparing patients with and without T cell clonotype expansion (left 5 panels). Correlation between dormancy gene signature and number of expanded clonotypes for each patient (right panel). **F.** Average expression levels of *GZMB*, *GZMA*, *INF*γ, *TNF* in CD8 and CD4 T cells comparing patients with low and high dormancy signatures.

Supplementary Table 1. Primers for mouse genotyping.

Mouse Strain	Primer	Sequence
LysM-Cre	LysM-Cre-R	GTTGCATCGACCGGTAATGCA
	LysM-common-F	CTTGGGCTGCCAGAATTTCTC
	LysM-wt-R	TTACAGTCGGCCAGGCTGAC
Tgfbr2-floxed	Tgfbr2-flox-F	TAAACAAGGTCCGGAGCCCA
	Tgfbr2-flox-R	ACTTCTGCAAGAGGTCCCCT
rtTA-floxed	rtTA-F	TGCCGCCATTATTACGACAAGC
	rtTA-R	ACCGTACTCGTCAATTCCAAGGG
Tgfbr2-shRNA	Tgfbr2-shR-F	CCATGAAGATCAAGGTGGTCGA
	Tgfbr2-shR-R	CCGTCTTCGTATGTGGTGATTCT

Supplementary Table 2. The list of anti-mouse antibodies used in CyTEK12

Antigen	Cat #	Clones	Company
CD45	564279	30-F11	BD biosciences
CD11b	612977	M1/70	BD biosciences
CD11c	117310	N418	Biolegend
CD206	141714	C068C2	Biolegend
F4/80	749283	T45-2342	BD biosciences
Ly-6G	127629	1A8	Biolegend
Ly6C	128036	HK1.4	Biolegend
PDL1	563369	MIH5	BD biosciences
iNOS	12-5920-82	CXNFT	eBioscience
Arg1	17-3697-82	A1exF5	eBioscience
CD103	121408	2E7	Biolegend
CD200	565547	OX-90	BD biosciences
CD200R	566345	OX-110	BD biosciences
B220	751580	RA3-6B2	BD biosciences
CD40	124618	3/23	Biolegend
CD44	103056	IM7	Biolegend
CD62L	104410	MEL-14	Biolegend
CD3	100249	17A2	Biolegend
CD4	553043	RM4-5	BD biosciences
CD25	102004	PC61	Biolegend
CD8a	612898	53-6.7	BD biosciences
FoXP3	126406	MF-14	Biolegend
IFNg	505830	XMG1.2	Biolegend
IL-6	561376	MP5-20F3	BD biosciences
TNFa	506338	MP6-XT22	Biolegend
Tbet	561263	O4-46	BD biosciences
TIM3	134012	B8.2C12	Biolegend
PD1	109112	RMP1-30	Biolegend
NK1.1	560618	PK136	BD biosciences
Perforin	154306	S16009A	Biolegend
TCR γ/δ	118124	GL3	Biolegend
IL-2	503824	JES6-5H4	Biolegend
IL-17A	506927	TC11-18H10.1	Biolegend
IL-2	503824	JES6-5H4	Biolegend
IL-13	159403	W17010B	Biolegend
IL-10	563277	JES5-16E3	BD biosciences
Granzyme B	515406	GB11	Biolegend
CD80	46-0801-82	16-10A1	Bioscience
CD86	105027	GL-1	Biolegend
Ki67	556027	B56	BD biosciences
CD317	127105	129C1	Biolegend
pFAK (Y397)	ab81298	EP2160Y	abcam
pERK (T202, Y204)	560115	20A	BD biosciences
p-p38 (T180, Y182)	12-9078-42	4NIT4KK	Invitrogen

Supplementary Table 3. The list of anti-mouse Antibodies using in IBEX

Antigen	Cat #	Clones	Company
CD45	58-0451-82	30-F11	Invitrogen
CD11b	101217	M1/70	Biolegend
F4/80	41-4801-82		Invitrogen
Ly6G	46-9668-82	1A8	Invitrogen
CD11c	117346		biolegend
CD3	100282	17A2	Biolegend
CD8	100708	53-6.7	Biolegend
CD103	MAB1990-SP	262523	R&D
CD200R	566345	OX-110	BD biosciences
Cl-Caspase3	D3E9		CST
Ki67	48-5698-82		Invitrogen
FoXP3	50-5773-82	FJK-16s	Invitrogen
NFAT	(D43B1) XP ®		CST
CD4	41-0042-82	RM4-5	Invitrogen
NKp46	AF2225		R&D
HMGB1	651406	3E8	Biolegend
Goat IgG	A11055		Invitrogen
Rabbit IgG	A31573		Invitrogen
Hoecht	40046		Biotium

Supplementary Table 4. The list of RT-qPCR primers

Target gene	Forward Primer (5’ to 3’)	Reverse Primer (3’ to 5’)
mSlurp1	GGTCACGGAAGCAACAGAAG	GGCCTTCCGATGCTATACCT
mKlf4	TGCCAGACCAGATGCAGTCAC	GTAGTGCCTGGTCAGTTCATC
mCd200	ACAGCCCATAGTACACCTTCA	TGTCCCAGTACCCTTCCAGG
mIfngr1	TACAGGTAAAGGTGTATTCGGGT	ACCGTGCATAGTCAGATTCTTTT
mGapdh	AATGTGTCCGTCGTGGATCTGA	GATGCCTGCTTCACCACCTTCT

Supplementary Table 5. The list of shRNA sequences

shRNA	Catalog #	shRNA sequence
shScrambled	TRCN0000190210	shRNA Control Plasmid
shSlurp1 #1	TRCN0000190210	CCTGTAAGACTGTACTGGAGA
shSlurp1 #2	TRCN0000190254	CTTCCGATGCTATACCTGTGA
shSlurp1 #3	TRCN0000189776	GAAGACACAGCCTGTAAGACT
shIfngr1 #1	TRCN0000067368	CCACATAGAATATCAGACTTA
shIfngr1 #2	TRCN0000067369	GCCAGAGTTAAAGCTAAGGTT
shIfngr1 #3	TRCN0000067371	CCACACTGGATTCCAGATATT
shCd200 #1	TRCN0000066679	GCCCATAGTACACCTTCACTA
shCd200 #2	TRCN0000066681	CGAGAGTCACTTCCATTCAAA
shCd200 #3	TRCN0000066682	CAGAGTCTGGACAAAGGATTT
shCd200 #4	TRCN0000066678	CCTGCCTACAAAGACAGGATA
shKlf4 #1	TRCN0000095370	CTCTCTCACATGAAGCGACTT
shKlf4 #2	TRCN0000095371	CTGGACCTAGACTTTATCCTT

Supplementary Table 6. The list of ChIP qPCR Primers

Slurp1 promoter region	Forward Primer (5' to 3')	Reverse Primer (3' to 5')
-850 ~ -600	CCATCCACAGGGCCACTCAT	GTCCTGACAGCAGAACTCTACCT
-500 ~ -250	CAGGTACTCCCTCCTTTCCATACTG	ACTGAGGAAGCCTTTTAGAGCC
-150 ~ +30	GGCCCCACCCTGGGATGGTAGGTGA	TCTTCAGTGCTCAGGAGCTAGGA

Cd200 promoter region	Forward Primer (5' to 3')	Reverse Primer (3' to 5')
-200 - +30	CTACGTCACCCTATACTGCCATTTGG	AGGCAGACTCTACAGCTCCTCTAGTG
-500 - -300	CAGGTACTCCCTCCTTTCCATACTG	ACTGAGGAAGCCTTTTAGAGCC
-800 to -600	GGCCCCACCCTGGGATGGTAGGTGA	TCTTCAGTGCTCAGGAGCTAGGA
-1100 to -900	GAATACCTTCTCACACCAGAGAGACTAG	TCGAGAAAGAGAGAGAGAGGAGAGAG

Supplementary Table 7. Slurp1 sgRNA target sequences and PCR primer sequences used in Slurp1 KO validation

**Name**	**Forward sequence (5' to 3')**	**Reverse Sequence (3' to 5')**
Slurp1 sgRNA #1	CCATCCACAGGGCCACTCAT	GTCCTGACAGCAGAACTCTACCT
Slurp1 PCR #1	GCCAGGCTCTAAAAGGCTT	CTGTCTCCAGTACAGTCTTAC
Slurp1 PCR #2	GACAGCAGAGCATGGTGTC	GTCTTCCATCTTGCACTGAG
